# Supplementary material for: Mental health in Germany before, during and after the COVID-19 pandemic
Source: PLoS One. 2025 Jan 3;20(1):e0313689. doi: 10.1371/journal.pone.0313689 (PMC11698453; doi:10.1371/journal.pone.0313689)
Supplement: S1 Appendix — (PDF) [file pone.0313689.s001.pdf]

Supporting Information

Mental health in Germany before, during and after the COVID-19 pandemic

Alexander Patzina, Matthias Collischon, Rasmus Hoffmann, Maksym Obrizan

Replication file can be found here: https://doi.org/10.1101/2024.06.21.24309286

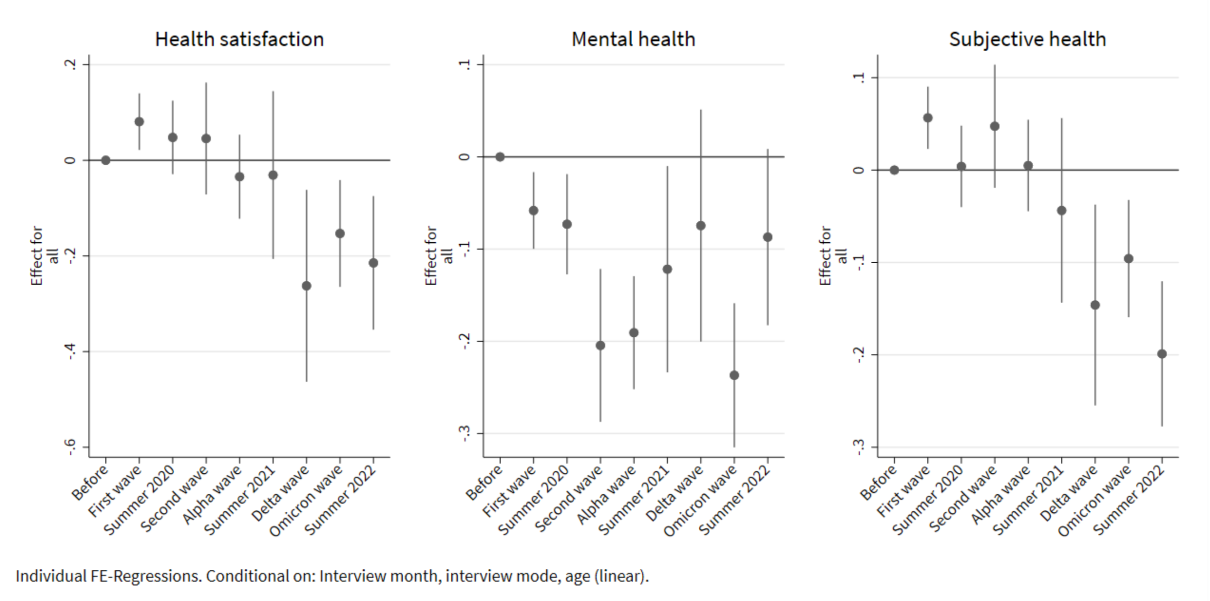

S1 Fig. Effects on other outcomes. Note: Figure shows point estimates of the employed time variable based on individual fixed effects regressions. Control variables: Interview month, interview mode, and age (linear). The left-hand side of this figure shows the results of health satisfaction measured with a single-item question ("How satisfied are you today with the following areas of your life and ? How satisfied are you with your health?") ranging from 0 to 10. The middle part of this figure shows the results for mental health problems ("How strongly have you been affected by mental problems, such as fear, dejection or irritability in the past 4 weeks? Please tell me whether you were affected (1) 'Extremely'; (2) 'Quite a bit'; (3) 'Moderately'; (4) 'A little bit'; (5) 'Not at all'") ranging from 1 to 5. The right-hand side of this figure shows the results for self-rated health ("How would you describe your state of health in the past 4 weeks in general? Was it (1) 'Bad'; (2) 'Not so good'; (3) 'Satisfactory', (4) 'Good'; (5) 'Very good'") ranging from 1 to 5.

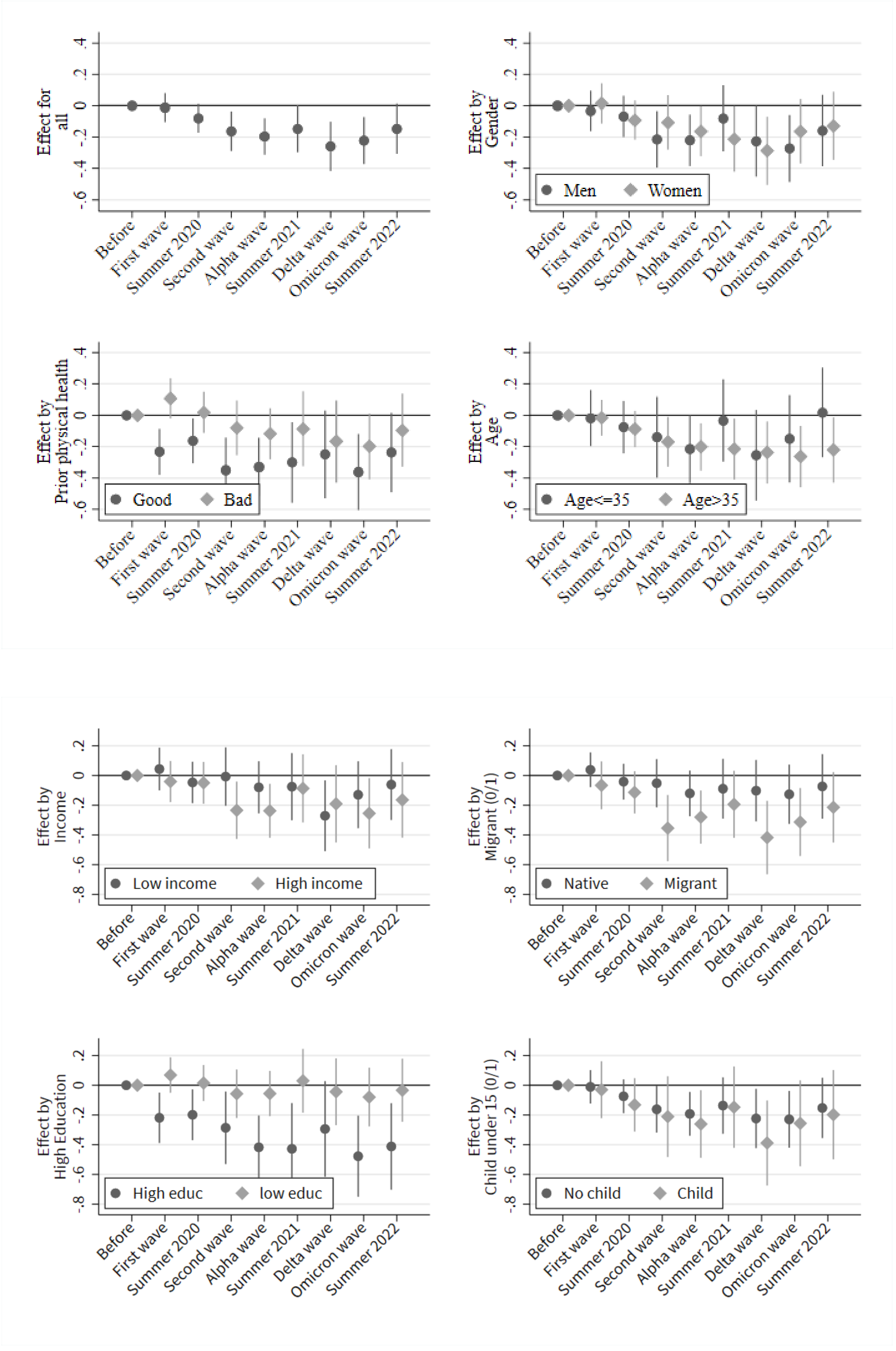

S2 Fig. Effects on psychological well-being.

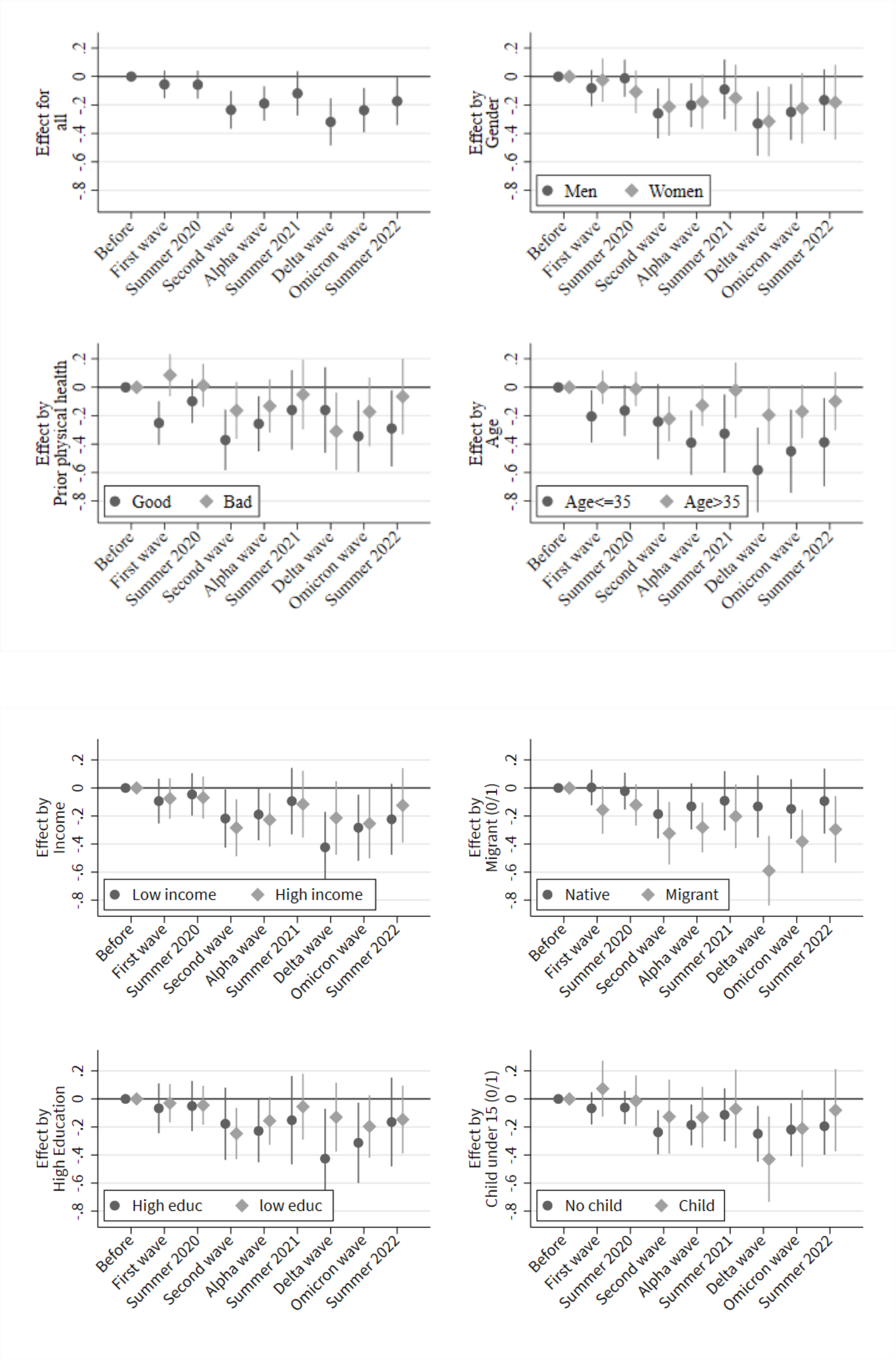

S3 Fig. Effects on feeling calm.

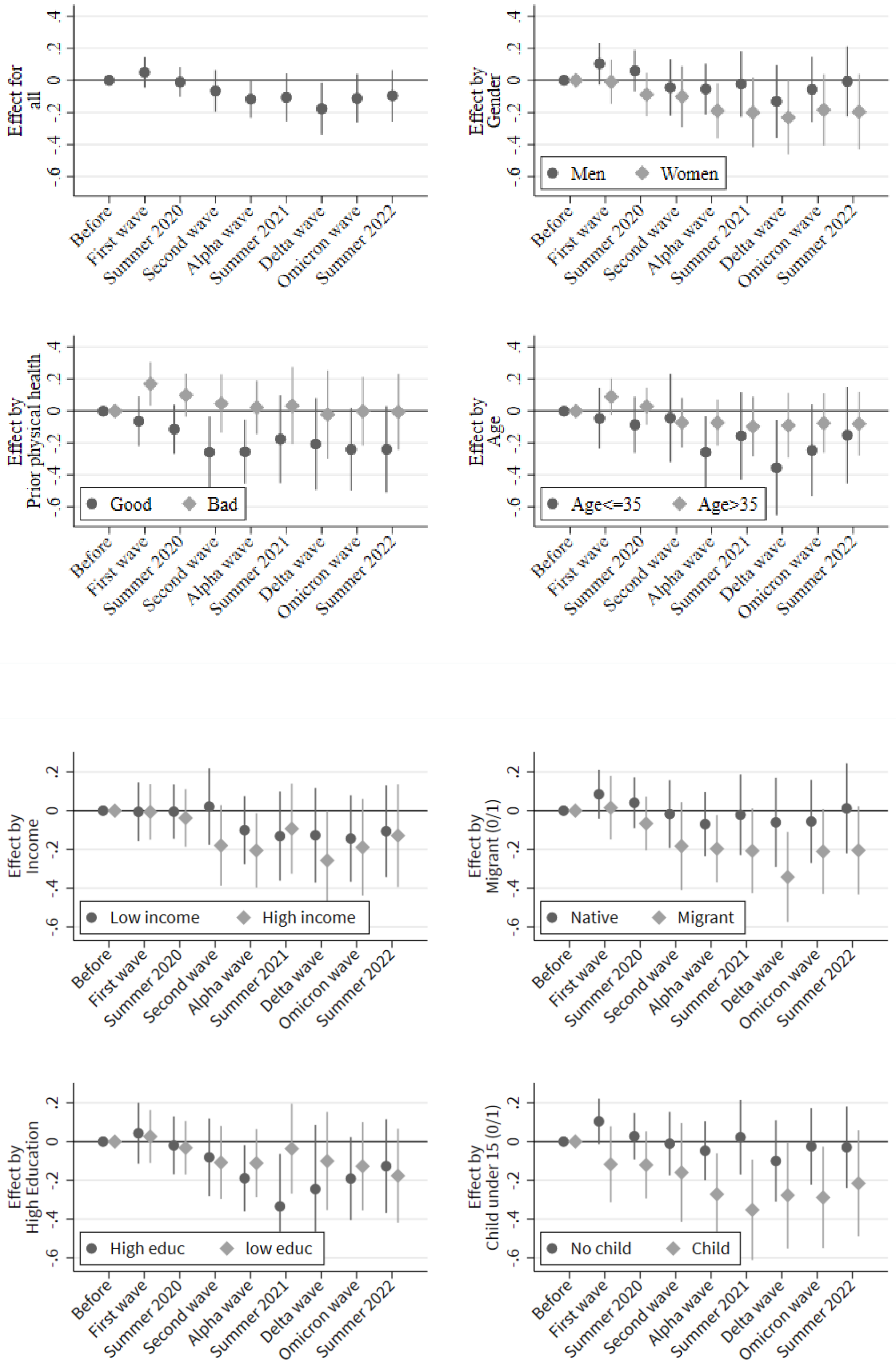

S4 Fig. Effects on vitality.

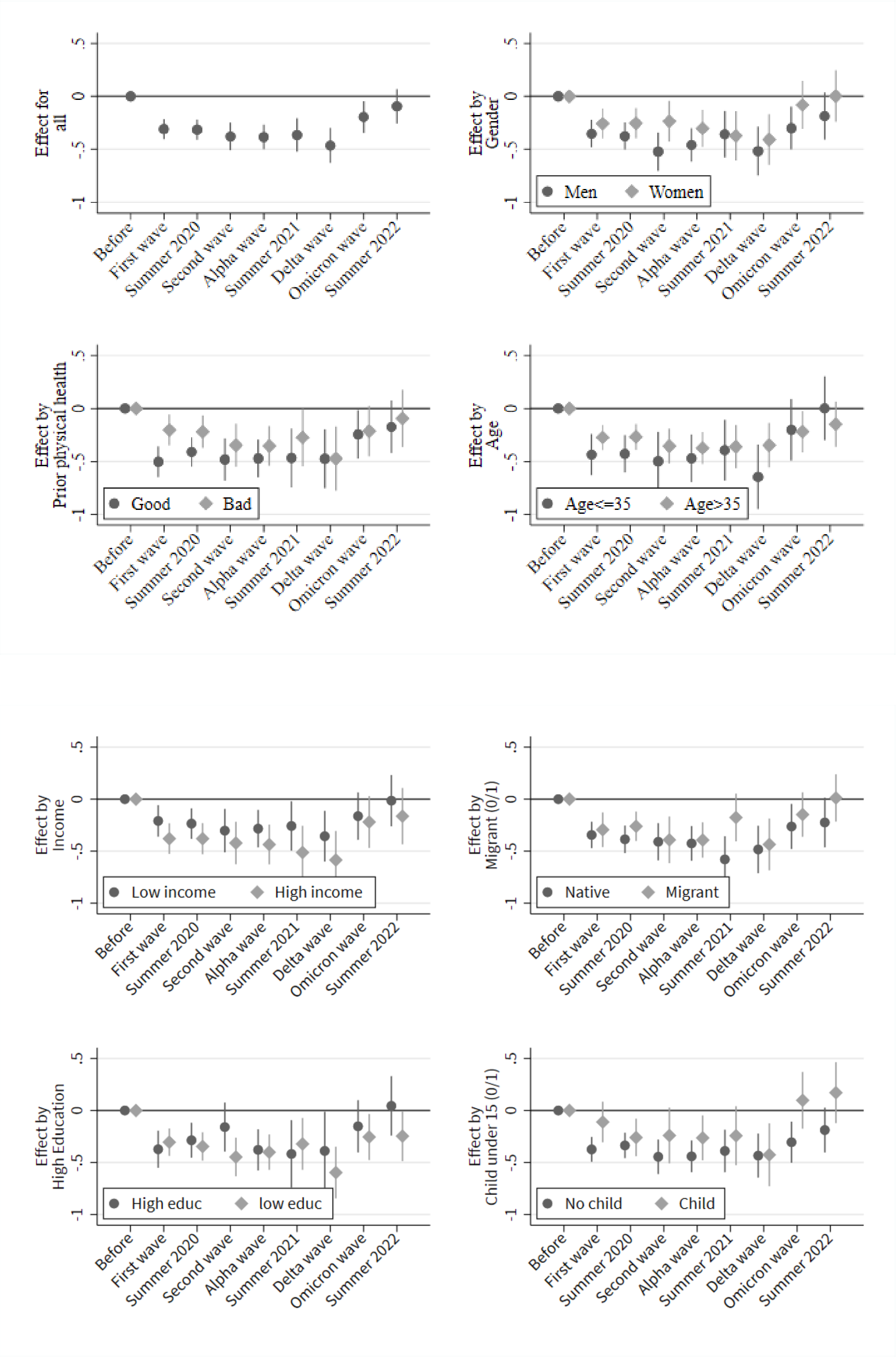

S5 Fig. Effects on emotional stability.

S1 Table. Results corresponding to Figure 3.

	(1) Main	(2) Men	(3) Women	(4) Good health	(5) Bad health	(6) Age <35	(7) Age >=35
**Pandemic phase**							
First wave (baseline: before)	-0.081*	-0.089*	-0.070	-0.264***	0.048	-0.178**	-0.048
	(0.032)	(0.043)	(0.049)	(0.050)	(0.048)	(0.061)	(0.040)
Summer 2020	-0.114***	-0.097*	-0.134**	-0.197***	-0.016	-0.186**	-0.083*
	(0.032)	(0.043)	(0.049)	(0.049)	(0.049)	(0.057)	(0.041)
Second wave	-0.212***	-0.265***	-0.163*	-0.369***	-0.131*	-0.242**	-0.205***
	(0.044)	(0.060)	(0.066)	(0.071)	(0.066)	(0.088)	(0.055)
Alpha wave	-0.221***	-0.233***	-0.209***	-0.328***	-0.139*	-0.334***	-0.194***
	(0.040)	(0.053)	(0.060)	(0.063)	(0.061)	(0.071)	(0.051)
Summer 2021	-0.179***	-0.132	-0.229**	-0.278**	-0.077	-0.223*	-0.168*
	(0.052)	(0.071)	(0.076)	(0.089)	(0.085)	(0.088)	(0.067)
Delta wave	-0.299***	-0.291***	-0.308***	-0.279**	-0.224*	-0.452***	-0.211**
	(0.056)	(0.075)	(0.084)	(0.097)	(0.100)	(0.098)	(0.070)
Omicron wave	-0.191***	-0.221**	-0.162*	-0.299***	-0.138	-0.264**	-0.182**
	(0.051)	(0.068)	(0.078)	(0.082)	(0.078)	(0.091)	(0.065)
Summer 2022	-0.125*	-0.130	-0.119	-0.234**	-0.055	-0.128	-0.134
	(0.055)	(0.074)	(0.083)	(0.087)	(0.086)	(0.095)	(0.070)
**Interview month**							
March (baseline:	-0.020	-0.013	-0.027	-0.023	-0.033	0.067	-0.040*
February)	(0.017)	(0.024)	(0.025)	(0.027)	(0.024)	(0.044)	(0.019)
April	-0.008	-0.014	-0.004	0.005	-0.026	0.090	-0.035
	(0.020)	(0.027)	(0.028)	(0.031)	(0.028)	(0.047)	(0.022)
May	-0.023	-0.042	-0.005	-0.029	-0.041	0.060	-0.049*
	(0.021)	(0.029)	(0.031)	(0.033)	(0.031)	(0.049)	(0.024)
June	-0.026	-0.071*	0.019	-0.085*	-0.042	0.073	-0.059
	(0.026)	(0.036)	(0.038)	(0.040)	(0.040)	(0.056)	(0.032)

July	0.007	-0.043	0.059	-0.009	-0.047	0.098	-0.034
	(0.029)	(0.039)	(0.043)	(0.043)	(0.044)	(0.059)	(0.036)
August	-0.014	-0.051	0.025	-0.060	-0.054	0.071	-0.048
	(0.031)	(0.043)	(0.046)	(0.049)	(0.049)	(0.064)	(0.039)
September	-0.003	-0.018	0.013	-0.040	-0.016	0.110	-0.053
	(0.036)	(0.050)	(0.054)	(0.058)	(0.061)	(0.071)	(0.045)
**Interview mode**							
CAPI, face-to-face (baseline: CATI)	-0.003	-0.017	0.009	0.044	-0.032	-0.028	0.009
	(0.019)	(0.026)	(0.028)	(0.028)	(0.027)	(0.038)	(0.023)
CAPI, phone	0.023	0.008	0.040	0.022	0.039	0.022	0.036
	(0.015)	(0.021)	(0.022)	(0.024)	(0.024)	(0.030)	(0.019)
Age (years)	0.015	0.022	0.009	0.007	0.021	0.023	0.016
	(0.012)	(0.016)	(0.019)	(0.020)	(0.019)	(0.021)	(0.016)
Constant	2.929***	2.755***	3.126***	3.757***	2.294*	3.012***	2.773***
	(0.542)	(0.715)	(0.838)	(0.851)	(0.937)	(0.530)	(0.841)
Observations	40020	20058	19956	13158	17565	12534	27486
Individuals	18704	9608	9100	5793	7171	7305	12055

S1 Table. Results corresponding to Figure 3 (cont).

	(1) Low income	(2) High income	(3) Native	(4) Migrant	(5) High education	(6) Low education	(7) No child	(8) Children
**Pandemic phase**								
First wave (baseline: before)	-0.062	-0.121*	-0.053	-0.126*	-0.055	-0.153**	-0.085*	-0.046
	(0.049)	(0.050)	(0.044)	(0.056)	(0.046)	(0.053)	(0.040)	(0.067)
Summer 2020	-0.081	-0.131*	-0.100*	-0.139**	-0.097*	-0.140**	-0.110**	-0.130*
	(0.047)	(0.052)	(0.046)	(0.047)	(0.047)	(0.052)	(0.041)	(0.061)
Second wave	-0.132*	-0.275***	-0.163**	-0.322***	-0.212***	-0.177*	-0.216***	-0.183*
	(0.065)	(0.071)	(0.061)	(0.076)	(0.063)	(0.074)	(0.056)	(0.090)
Alpha wave	-0.164**	-0.272***	-0.184**	-0.290***	-0.175**	-0.304***	-0.217***	-0.229**
	(0.057)	(0.066)	(0.058)	(0.056)	(0.059)	(0.061)	(0.052)	(0.074)
Summer 2021	-0.134	-0.198*	-0.192**	-0.190**	-0.086	-0.325***	-0.150*	-0.196*
	(0.074)	(0.082)	(0.074)	(0.073)	(0.079)	(0.096)	(0.066)	(0.092)
Delta wave	-0.287***	-0.307**	-0.192*	-0.440***	-0.204*	-0.339**	-0.249***	-0.371***
	(0.081)	(0.096)	(0.079)	(0.080)	(0.085)	(0.122)	(0.070)	(0.103)
Omicron wave	-0.181*	-0.224**	-0.146	-0.266***	-0.158*	-0.285***	-0.196**	-0.161
	(0.072)	(0.086)	(0.075)	(0.070)	(0.076)	(0.076)	(0.067)	(0.092)
Summer 2022	-0.097	-0.140	-0.092	-0.173*	-0.143	-0.157	-0.140*	-0.073
	(0.077)	(0.092)	(0.082)	(0.074)	(0.081)	(0.087)	(0.071)	(0.099)
**Interview month**								
March (baseline:	0.000	-0.047*	-0.018	-0.030	-0.044*	0.059	-0.028	0.012
February)	(0.028)	(0.024)	(0.019)	(0.042)	(0.021)	(0.038)	(0.019)	(0.047)
April	0.013	-0.019	-0.000	-0.046	-0.021	0.059	-0.023	0.038

	(0.033)	(0.027)	(0.022)	(0.046)	(0.024)	(0.042)	(0.022)	(0.051)
May	-0.038	-0.015	-0.011	-0.065	-0.026	-0.006	-0.032	0.016
	(0.034)	(0.030)	(0.025)	(0.046)	(0.026)	(0.046)	(0.024)	(0.051)
June	-0.004	-0.080^{*}	-0.014	-0.066	-0.029	-0.029	-0.043	0.045
	(0.042)	(0.038)	(0.032)	(0.052)	(0.034)	(0.054)	(0.031)	(0.059)
July	-0.050	0.036	0.041	-0.057	-0.022	0.082	0.002	0.052
	(0.046)	(0.042)	(0.036)	(0.055)	(0.038)	(0.058)	(0.035)	(0.064)
August	0.023	-0.071	-0.005	-0.063	-0.028	0.037	-0.051	0.074
	(0.050)	(0.046)	(0.040)	(0.059)	(0.042)	(0.066)	(0.037)	(0.069)
September	-0.000	-0.067	0.013	-0.059	0.005	-0.029	-0.020	0.055
	(0.055)	(0.058)	(0.048)	(0.064)	(0.051)	(0.086)	(0.044)	(0.077)
**Interview mode**								
CAPI, face-to-face (baseline: CATI)	-0.005	0.007	0.001	-0.009	-0.010	0.014	-0.016	0.011
	(0.030)	(0.028)	(0.024)	(0.033)	(0.025)	(0.039)	(0.023)	(0.040)
CAPI, phone	-0.000	0.042	0.024	0.044	0.028	0.008	0.018	0.056
	(0.024)	(0.023)	(0.020)	(0.027)	(0.020)	(0.033)	(0.019)	(0.030)
Age (years)	0.020	0.017	0.006	0.023	0.007	0.036^{*}	0.017	0.000
	(0.017)	(0.021)	(0.018)	(0.016)	(0.018)	(0.017)	(0.016)	(0.021)
Constant	2.582^{***}	2.993^{**}	3.281^{***}	2.815^{***}	3.253^{***}	1.978^{*}	2.774^{***}	3.629^{***}
	(0.721)	(0.956)	(0.870)	(0.623)	(0.891)	(0.825)	(0.766)	(0.748)
Observations	20305	19237	25216	14010	22097	6026	29633	10387
Individuals	11935	9207	10730	7609	8433	2122	13809	5750

Note: Pass data. Standard errors in parentheses. * $p < 0.05$, ** $p < 0.01$, *** $p < 0.001$.

S2 Table. Results corresponding to Figure 4.

	(1) Psychological Well-being	(2) Feeling Calm	(3) Vitality	(4) Role emotional
**Pandemic phase**				
First wave (baseline: before)	-0.013	-0.055	0.050	-0.309***
	(0.047)	(0.050)	(0.048)	(0.049)
Summer 2020	-0.080	-0.057	-0.010	-0.315***
	(0.047)	(0.050)	(0.048)	(0.049)
Second wave	-0.162*	-0.235***	-0.066	-0.379***
	(0.065)	(0.068)	(0.066)	(0.067)
Alpha wave	-0.196**	-0.190**	-0.117*	-0.384***
	(0.060)	(0.062)	(0.060)	(0.060)
Summer 2021	-0.147	-0.119	-0.107	-0.365***
	(0.076)	(0.080)	(0.076)	(0.081)
Delta wave	-0.259**	-0.319***	-0.177*	-0.465***
	(0.081)	(0.085)	(0.083)	(0.085)
Omicron wave	-0.222**	-0.237**	-0.113	-0.195*
	(0.077)	(0.080)	(0.077)	(0.077)
Summer 2022	-0.147	-0.173*	-0.097	-0.094
	(0.082)	(0.086)	(0.082)	(0.083)
**Interview month**				
March (baseline:	-0.010	-0.043	0.003	-0.027
February)	(0.023)	(0.026)	(0.025)	(0.025)
April	-0.026	-0.037	0.036	0.001
	(0.026)	(0.029)	(0.028)	(0.029)
May	-0.016	-0.053	0.030	-0.047
	(0.029)	(0.031)	(0.031)	(0.031)
June	-0.025	-0.064	0.058	-0.068
	(0.036)	(0.038)	(0.038)	(0.039)

July	0.032	-0.019	0.079	-0.045
	(0.040)	(0.043)	(0.042)	(0.043)
August	0.007	-0.064	0.059	-0.042
	(0.043)	(0.046)	(0.047)	(0.047)
September	-0.008	-0.059	0.119*	-0.051
	(0.051)	(0.056)	(0.054)	(0.057)
**Interview mode**				
CAPI, face-to-face	0.064*	-0.086**	-0.063*	0.063*
(baseline: CATI)	(0.026)	(0.028)	(0.028)	(0.028)
CAPI, phone	0.062**	-0.012	-0.032	0.069**
	(0.021)	(0.023)	(0.023)	(0.024)
Age (years)	0.017	0.020	0.006	0.017
	(0.019)	(0.019)	(0.019)	(0.019)
Constant	2.923***	2.641**	2.805***	3.412***
	(0.825)	(0.854)	(0.822)	(0.824)
Observations	40285	40268	40223	40146
Individuals	18833	18825	18803	18755

Note: Pass data. Standard errors in parentheses. * $p < 0.05$, ** $p < 0.01$, *** $p < 0.001$.
